# Evaluating the link between periodontitis and oral squamous cell carcinoma through Wnt/β-catenin pathway: a critical review

**DOI:** 10.3389/froh.2025.1575721

**Published:** 2025-05-12

**Authors:** Montserrat Reyes, Hery Urra, Daniel Peña-Oyarzún

**Affiliations:** ^1^ Pathology and Oral Medicine Department, Faculty of Odontology, Universidad de Chile, Santiago, Chile; ^2^ School of Odontology, Faculty of Odontology, Universidad San Sebastián, Santiago, Chile

**Keywords:** oral squamous cell carcinoma (OSCC), periodontitis, Wnt/β-catenin signaling, leukoplakia, transcriptional expression

## Abstract

Oral Squamous Cell Carcinoma (OSCC), the main form of oral cancer, is a major health problem globally that affects 400,000 people every year. It has been postulated that periodontitis, a chronic inflammatory disease characterized by alveolar bone resorption, is an independent risk factor for OSCC. However, the mechanisms underlying this link are not fully elucidated. It has been demonstrated that the Wnt/β-catenin pathway is key to the transformation of oral potentially malignant disorders (OPMD) towards OSCC (i.e., leukoplakia), particularly in OPMD histologically diagnosed as oral dysplasia. Using a GEO database of oral carcinogenesis (GSE85195), the transcriptional modification of 19 Wnt ligands and 4 key regulatory proteins of β-catenin, including E-cadherin, APC, AXIN and GSK3B, during leukoplakia, and early and late stages OSCC, was determined. The transcriptional expression of these targets was also assessed in periodontitis (GEO database GSE223924). Together, it was found that Wnt ligands Wnt3, Wnt3a, Wnt5b and Wnt7b are concomitantly upregulated in periodontitis and oral carcinogenesis. With these results, and the information retrieved from the literature, this review discusses the potential role of the Wnt/β-catenin pathway as a molecular mechanism that could interlink periodontitis and OSCC.

## 1 Introduction

Oral cancer, one of the most common forms of Head and Neck cancer, affects nearly 400,000 people per year, with an average survival rate after five years of 50% (1). Oral cancer occurs more frequently at the floor and the anterior base of the mouth, and at the dorsal surface of the tongue, but can also present at the lips, the gums, the hard palate mucosa and the soft palate mucosa (1). Oral cancer usually evolves from an oral potentially malignant disorder (OPMD), such as leukoplakia and erythroplakia, among others (2). These lesions appear as non-healing ulcers with white or red patches and indurated and irregular margins (2).

Malignant transformation occurs in almost 80% of OPMDs histologically diagnosed as oral dysplasia and sustained pro-inflammatory oral microenvironment seems to promote this transformation (2). Indeed, cytokines that engage nuclear factor kappa B (NFKB) signaling like tumor necrosis factor alpha (TNFA/TNF-α), Interleukin-1 (IL1), IL6 and IL8, are increased in saliva during OPMDs (3). These pro-inflammatory cytokines are associated with increased oral cancer burden and aggressiveness (3). Similarly, a case-control study shows that inflammatory oral mucosa conditions, including poor dental hygiene, oral graft vs. host disease and glossitis, are directly correlated with a predisposition to develop oral cancer from OPMDs (4). Besides tobacco smoking and alcohol consumption, it has been proposed recently that periodontitis, a chronic inflammatory disease of the gum pocket surrounding the teeth, is an independent risk factor for developing oral cancer (5).

We and others have identified the Wnt/β-catenin signaling pathway as a key driver for malignant transformation of OPMDs with oral dysplasia (6). Despite increased Wnt/β-catenin signaling has been reported in periodontitis (7), the possible role of this pathway during oral carcinogenesis associated with periodontitis has not been exhaustively explored. In this review we summarize the current information regarding the role of the Wnt/β-catenin signaling pathway in the most predominant type of oral cancer, the oral squamous cell carcinoma (OSCC), and periodontitis. We also evaluated the mRNA expression levels of several Wnt ligands and other proteins involved in β-catenin stabilization and activation, such as E-cadherin, AXIN, APC and GSK3B using a GEO database for periodontitis (GSE223924) and oral carcinogenesis (GSE85195). Combining the studies available in literature and our GEO database results, we shed light on the link between periodontitis and OSCC, by activation of the Wnt/β-catenin pathway.

## 2 Oral squamous cell carcinoma (OSCC)

OSCC is an epithelial malignancy occurring in the oral mucosa, mainly due to cigarette smoking, alcohol consumption or human papilloma virus (HPV) infection (8). OSCC is commonly preceded by an OPMD, that in most cases are histologically diagnosed as epithelial dysplasia (2). During an oral epithelial dysplasia, cells present atypia (i.e., increased nucleus/cytoplasm ratio, augmented nucleolus number, cell shape changes, among others) in few or multiple epithelial layers, suggesting abnormal proliferation and maturation (i.e., irregular stratification and loss of basal cell polarity, among others) (9). According to the percentage of epithelial layers compromised, oral epithelial dysplasia could be classified as low-grade dysplasia (previously known as mild dysplasia), or high-grade dysplasia (previously known as moderate or severe dysplasia) (2). Once the OSCC is established, a subepithelial desmoplastic reaction and immune infiltration is observed, enhancing the proliferative, migratory and invasive capacity of OSCC cells, which metastasize predominantly to the lungs, liver and bones (9). Thus, tumor/stroma ratio, depth of invasion and extracapsular spread are important for OSCC TNM grading and patient prognostic (9).

Exposure to OSCC risk factors, aggravated by aging, provoke dysregulations in the oral mucosa, including immune suppression, autocrine activation of cancer-associated fibroblasts, hypoxic activation of transcriptional factors, downregulation of the tumor suppressor proteins TP53 and RB1 and upregulation of signaling pathways that induce survival and proliferation of OSCC cells (10, 11). Among these pathways, the Wnt/β-catenin pathway is the most studied in OPMD preceding the establishment of OSCC, underscoring its role during OSCC carcinogenesis (10). Consistently, in OSCC carcinogenesis *in vivo* models, where mice are challenged with the tobacco mimetic 4-Nitroquinoline 1-oxide (4NQO), suppression of Wnt/β-catenin activity with C59 compound reduces the size, the number and the grading of the dysplastic and OSCC lesions (6).

## 3 Periodontitis

Periodontitis is a chronic inflammation of the periodontal tissue. Diagnostic separate into mild periodontitis when probe depth of the gingival sulcus (which is used for clinical attachment loss calculation) is between 4 and 5 mm, as moderate periodontitis when probe depth is between 6 and 7 mm, and as severe periodontitis when probe depth is over 7 mm (12). During periodontitis a bacterial biofilm is formed around the tooth, causing persistent inflammation of the gingival tissue, ultimately leading to alveolar bone resorption (13). Pathogenic bacteria composing this biofilm are mostly anaerobic and gram-negative, like *Porphyromonas gingivalis*, *Fusobacterium nucleatum* and *Aggregatibacter actinomycetemcomitans*, among others (13). The migration of these bacteria into the subgingival space disrupts the oral mucosa epithelium by degrading the keratinocyte cell-to-cell contacts with gingipains and collagenases, while inducing an exacerbated inflammatory response (13). This inflammatory response is characterized by increased superoxide production as a result of overactivation of the nicotinamide adenine dinucleotide phosphate (NADPH) oxidase 4 (NOX4), downregulation of antioxidant transcription factor NFE2 like bZIP transcription factor 2 (NFE2L2/NRF-2), and increased secretion of pro-inflammatory cytokines, such as C-C motif chemokine ligand 2 (CCL2/MCP-1), IL1B and IL6 (14).

Nearly 70% of the global population has some degree of periodontitis and its prevalence is age-dependent. The prevalence of severe periodontitis increases with age, being around 20% in adults between 35 and 44 years, and around 40% in adults over 60 years (12). Risk factors for periodontitis include smoking, diabetes mellitus, obesity, alcoholism, osteoporosis, and stress (12). Curiously, most of these are also risk factors for OSCC.

## 4 Periodontitis as an independent risk factor for OSCC

The idea that periodontitis is a risk factor for OSCC has been evaluated in a meta-analysis by Zeng and cols., where they find that periodontitis increases the Head and Neck Cancer risk -OSCC represents the 80% of Head and Neck Cancer cases- in 2.63 times (OR = 2.63, 95% CI = 1.68–4.14, *p* < 0.001) (15). These results, however, should be taken with caution, since meta-analysis included only 2 cohort and 6 case-control studies. Given the reduced sample size, high heterogeneity was observed among the studies. Similarly, five studies were included in a second meta-analysis, concluding that periodontitis promotes OSCC in 3.53 times (OR = 3.53, 95% CI = 1.52–8.23, *p* = 0.003) (16). This correlation is not exclusive for oral cancer, as hazard ratio for developing pancreatic (OR = 1.74, 95% CI = 1.21–2.52, *p* = 0.003), prostatic (OR = 1.25, 95% CI = 1.04–1.51, *p* = 0.02) and breast (OR = 1.11, 95% CI = 1.00–1.23, *p* = 0.04) cancer are also significantly increased when compared to patients without periodontitis (17). A similar correlation is observed when taking OSCC patients as the case population. Compared to patients without oral cancer, OSCC patients show higher clinical attachment loss (6.2 mm vs. 2.8 mm) and periodontal probe depth (5.6 mm vs. 2.5 mm), suggesting an elevated incidence of periodontitis in OSCC patients (18). Indeed, nearly 60% of OSCC patients have periodontitis. The correlation between OSCC and periodontitis is especially significant at late stages of periodontitis, since over 70% of patients with OSCC display stage IV periodontitis (18). It is important to note that this study cannot exclude a confounding factor given by the influence of age on OSCC and periodontitis. Additionally, a clinical case report shows that the presence of periodontitis in patients with OSCC also occurs in never-smokers, indicating that, at least in a fraction of OSCC patients, periodontitis and smoking are independent risk factors for OSCC (5).

Presence of periodontal pathogens has been reported in OSCC patients. *Fusobacterium*, *Pseudomonas*, *Campylobacter* and *Porphyromonas* species, involved in periodontitis, were identified in saliva samples by DNA sequencing (19). Moreover, levels of *Fusobacterium nucleatum* and *Porphyromonas gingivalis* are higher in OSCC lesions than in the contiguous oral mucosa (without apparent lesion) of the same patient (20). Similar abundance of *Fusobacterium* and *Campylobacter* species has been observed in oral leukoplasia, an OPMD (21). Indeed, together with serum IL6, serum immunoglobulin G against *Porphyromonas gingivalis* is proposed as a progression marker from OPMD to OSCC (22). However, more studies are required to support that conclusion.

All the above-mentioned studies do not exclude the possibility that periodontitis is a consequence of OSCC. Studies describing how periodontal pathogens induce malignant features of OSCC cells *in vitro* have addressed this issue. Infection of OSCC cells with *Porphyromonas gingivalis* promote stemness by increased expression of CD44 and CD133, as well as migration and invasion, suggesting that *Porphyromonas gingivalis* augments aggressiveness of OSCC cells (23). Injection of OSC-20 cell, an *in vitro* model of OSCC, infected with *Porphyromonas gingivalis* into mice shows a dramatic increase in the number of metastatic nodules (24). This effect was observed even after treatment with the chemotherapeutic taxol, a type of taxane, suggesting that *Porphyromonas gingivalis* participates during oral cancer chemoresistance (24). The gingipains present in *Porphyromonas gingivalis* can also lead to higher metastatic potential of OSCC cell lines by augmenting expression of matrix metallopeptidase 9 (MMP9) (23). MMP1 and MMP2 are increased by *Porphyromonas gingivalis* in an IL8-dependent manner (23). A pro-angiogenic IL1B signaling is elicited by *Porphyromonas gingivalis* and *Fusobacterium nucleatum*, where IL1B interacts with the vascular endothelial growth factor (VEGFR) (25). The lipopolysaccharide structure from these bacteria is sufficient to induce angiogenesis *in vitro* through activation of mitogen-activated protein kinase ERK1/2 (26). A metatranscriptomic analysis of OSCC tissues, compared to adjacent buccal sites, reveals that *Fusobacterium nucleatum* is associated to proteolysis, DNA mismatch repair, carbohydrate metabolism and citrate transport (27), while abundance of *Fusobacterium* species increases with OSCC staging (1.66% in stage 1, 2.41% in stages 2 and 3, and 3.31% in stage 4) (28). Thus, the effect of periodontal pathogens in models of oral carcinogenesis suggests a synergic role of periodontal infection in OSCC inflammation.

Studies on how periodontal pathogens may participate during oral carcinogenesis have also been performed *in vivo*. As mentioned, a well-known OSCC model *in vivo* is established using 4NQO. Mice treated with 4NQO and infected with *Porphyromonas gingivalis* displayed increased number and size of oral lesions, compared to mice treated with 4NQO alone (29, 30). Similar results are obtained after co-infection of 4NQO-treated mice with *Porphyromonas gingivalis* and *Fusobacterium nucleatum* (31). Interestingly, no differences in alveolar bone resorption are observed between mice treated with 4NQO and infected with *Porphyromonas gingivalis*, and mice only infected with *Porphyromonas gingivalis* (30). Together, this suggests that periodontitis features influence oral carcinogenesis, but not the other way around.

OSCC and periodontitis also share some epigenetic modifications in genes that are key for carcinogenesis. Similar hypermethylation pattern of miR193 CpG islands is present in oral brushing samples from OSCC and periodontitis patients (32). This suggests that reduced expression of the tumor suppressing gene miR193 due to hypermethylation may be an important feature to consider in patients with periodontitis (32, 33). Nevertheless, to assess the epigenetic modifications, authors recruited only 15 patients per condition so further studies would be required to reach a solid conclusion. A bioinformatic analysis showed that periodontitis and OSCC patients have 18 miRNAs with a similar trend (34). Those involved in carcinogenesis are hsa-mir-224, hsa-mir-210, hsa-mir-31, which are upregulated, and hsa-mir-497, hsa-mir-29c, hsa-mir-486, which are downregulated (34). Authors identified FBN1, HIF1A, TP53, E2F1, MYCN and JUN as the transcription factors that are concomitantly deregulated in periodontitis and OSCC, because of altered miRNA expression (34). Of these transcription factors, FBN1, HIF1A and MYCN are known to regulate Wnt/β-catenin pathway during different types of cancer, including lymphoma, hepatocellular carcinoma and neuroblastoma, among others (35).

## 5 Wnt/β-catenin signaling pathway in OSCC

Aberrant activation of the Wnt/β-catenin pathway has been associated with breast, gastrointestinal, and oral carcinogenesis, among others, as it promotes the expression of genes implicated in cell growth and death resistance (35). Canonically, to activate this pathway, an extracellular Wnt1 class ligand (i.e., Wnt1, Wnt2, Wnt2b, Wnt3, Wnt3a, Wnt8a, Wnt8b, Wnt10a, Wnt10b) binds to the 7-transmembrane domains receptor Frizzled (FDZ) and the LDL receptor related protein (LRP) 5/6 co-receptor, triggering the recruitment of dishevelled segment polarity protein 1 (DVL1) to FDZ through its Dishevelled Egl-10 and Pleckstrin (DEP) domain (36). This new membrane localization of DVL1 allows the sequestration and inactivation of a cytoplasmic complex designed to target β-catenin for proteasomal degradation (36). With this, β-catenin is no longer degraded and accumulates in the cytoplasm for nuclear translocation and transcription of TCF/LEF dependent genes, like MYC proto-oncogene, bHLH transcription factor (MYC), cyclin D1 (CCND1) and baculoviral IAP repeat containing 5 (BIRC5/Survivin) (35). Besides promoting proliferation, migration and invasion, activation of the Wnt/β-catenin also prevents the tumor infiltration of CD8^+^ cells, leading to poor OSCC patient survival (37). Under non-proliferative conditions, β-catenin forms a complex with E-cadherin at the cell membrane to form adherence junctions. Thus, loss of E-cadherin also promotes activation of the Wnt/β-catenin, a feature observed in many types of cancer (38).

**Figure 1:**
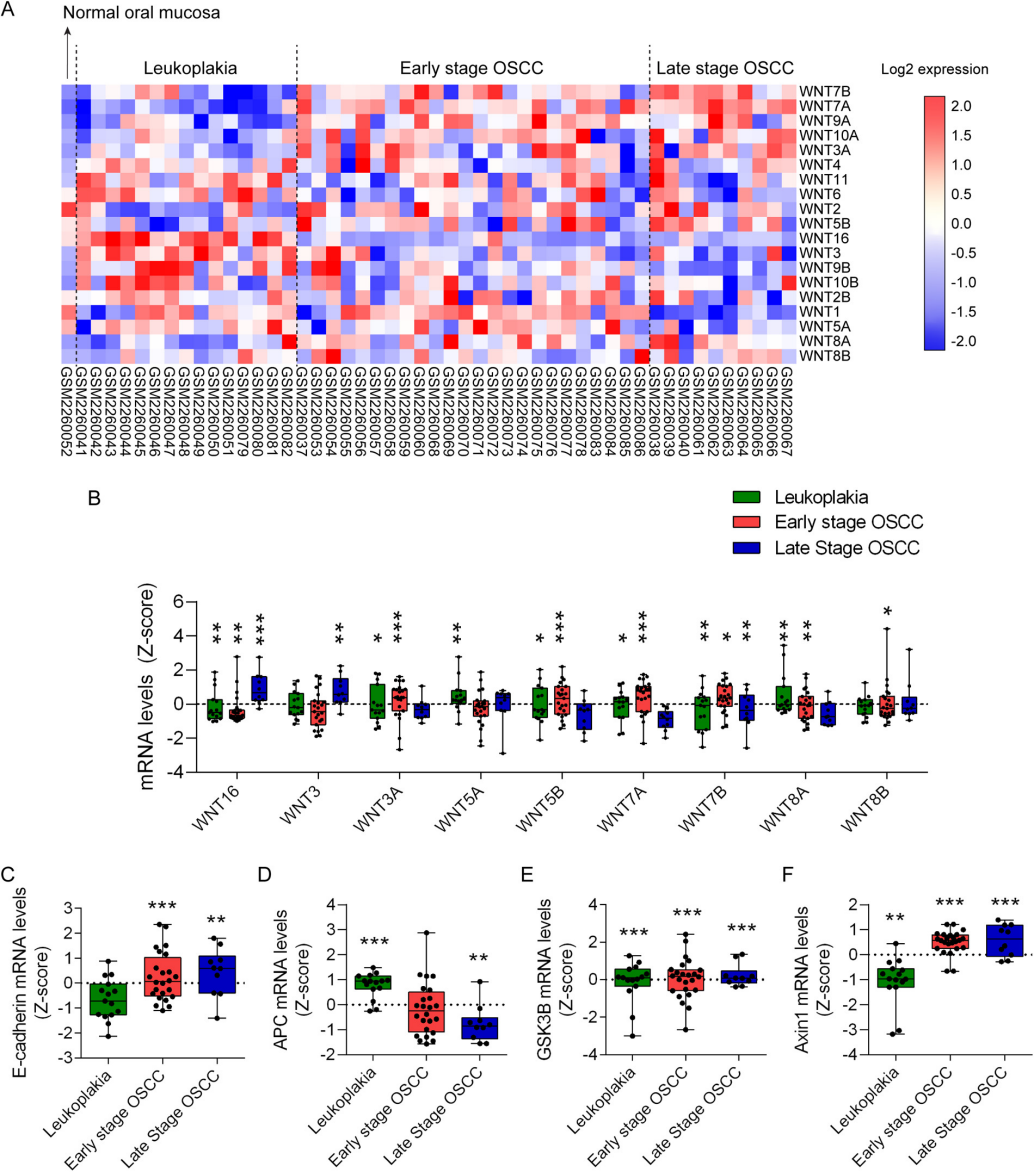
Transcriptomic analysis of Wnt ligands and components of β-catenin destruction complex in oral carcinogenesis. Transcriptomic datasets of 15 leukoplakia, 2 OSCC stage I, 22 OSCC stage II, 7 OSCC stage III and 3 OSCC stage IV samples were retrieved from the GEO database GSE85195. OSCC datasets were grouped as early stage (stages I and II) or late stage (stages II and IV). Only one normal oral mucosa was available in GSE85195. Raw transcriptomic data from the database were processed and normalized to *Z*-scores to standardize the data and allow direct comparison of expression levels. **(A)** Transcriptional Wnt ligand expression was organized in heat maps according to their expression patterns. **(B)** *Z*-scores of significantly upregulated Wnt ligands were graphed and arranged according to the Ward hierarchical clustering. One sample *t*-test was used for statistical analysis (*p < 0.05; **p < 0.01, ***p < 0.001, vs. the *Z*-score of each normal oral mucosa). Additionally, expression data for critical components of the Wnt signaling pathway, including **(C)** E-cadherin (CDH1), **(D)** APC, **(E)** GSK3B and **(F)** Axin1, were extracted and graphed using GraphPad Prism. One sample *t*-test was used for statistical analysis (**p < 0.01, ***p < 0.001, vs. the *Z*-score of each normal oral mucosa).

To explore the role of Wnt ligands in oral carcinogenesis, we analyzed the differential transcriptomic expression of Wnt ligands during OSCC and leukoplakia, the most frequent type of dysplasia-related OPMD, using the GEO database (GSE85195). Detailed information of the data retrieved is described in Supplementary methods. OSCC patients were classified according to their TNM staging as early stage (I or II stages) or late stage (III or IV stages) OSCC. Data from 15 patients with leukoplakia, 24 patients with early stage OSCC and 10 patients with late stage OSCC was retrieved. Only one normal oral mucosa was available as control. Binary logarithm mRNA expression of Wnt ligands was graphically displayed using heat maps (Figure 1A). Transcriptomic *Z*-score analysis shows significant upregulation of the canonical Wnt ligand Wnt3a (Figure 1B). Consistent with this, transcriptional and protein upregulation of Wnt3a in OSCC cell lines is observed after activation of the Hedgehog/Gli2 pathway (39). Additionally, activation of the Wnt/β-catenin pathway with exogenous Wnt3a augments CDK5RAP2, a protein involved in microtubule stabilization (40). By doing so, Wnt3a increases transcriptional expression of stemness-related genes in OSCC, suggesting that activation of Wnt/β-catenin in OSCC allows sustained cell renewal (40). Other studies show that increased immunohistochemical staining of Wnt3a in biopsies from patients is associated with higher grades of oral dysplasia and with poorer OSCC differentiation status (41). In dysplastic and OSCC cell lines, Wnt3a promotes nuclear accumulation of β-catenin, as well as cell migration and cell invasion (6). Wnt3a also controls the metabolic switch towards a glycolytic state of OSCC cells, which is required for their proliferation (42). However, the participation of β-catenin on this metabolic switch was not evaluated thoroughly, as increase in protein levels but not nuclear localization was determined (42). Our results from GEO database (GSE85195) also indicate that, compared to normal oral mucosa, Wnt3 mRNA levels are significantly increased in late stage OSCC (Figure 1B). A pathophysiological relevance of Wnt3 upregulation and Wnt/β-catenin pathway activation in OSCC cells is the acquisition of resistance against chemotherapeutic agents, like 5-Fluorouracil (43). However, how Wnt/β-catenin pathway induces chemoresistance in OSCC cells remains unknown. Finally, analysis of GEO database (GSE85195) also revealed that Wnt8a mRNA levels are significantly upregulated in both leukoplakia and early stage OSCC, while Wnt8b mRNA levels are significantly upregulated only in early stage OSCC (Figure 1B). To date, no studies have addressed whether Wnt8a or Wnt8b participates in oral malignancies.

Despite the role of canonic Wnt ligands in oral carcinogenesis previously described the contribution of the non-canonical Wnt signaling pathways, activated by Wnt5a class ligands (i.e., Wnt4, Wnt5a, Wnt5b, Wnt6, Wnt7a, Wnt7b and Wnt11), must not be excluded. Table 1 shows the classification of Wnt ligands according to pathway activation. Briefly, non-canonical Wnt signaling is divided into two branches: the Wnt/planar cell polarity (PCP) pathway and the Wnt/Ca^2+^ pathway (44). While the Wnt/PCP pathway promotes the complex formation between Dvl and the Dvl-associated activator of morphogenesis (DAAM) for RhoA-dependent modulation of actin polymerization, the Wnt/Ca^2+^ pathway follows activation of phospholipase C (PLC) and reticular Ca^2+^ release for protein kinase C (PKC)-dependent actin reorganization (44).

**Table 1:** Classification of Wnt ligands.

Canonic Wnt ligands	Non-canonic Wnt ligands
Wnt1	Wnt4
Wnt2	Wnt5a
Wnt2b	Wnt5b
Wnt3	Wnt6
Wnt3a	Wnt7a
Wnt8a	Wnt7b
Wnt8b	Wnt11
Wnt10a	
Wnt10b	

According to our transcriptomic analysis, significant upregulation of Wnt5b mRNA occurs during leukoplakia and early stage OSCC. Wnt5a mRNA is upregulated during leukoplakia (Figure 1B). Indeed, studies show that the use of tobacco mimetic 4NQO in rats induce expression of Wnt5a in pre-neoplastic lesions (45). This increases the metastatic potential of OSCC cells *in vitro* by upregulating MMP9 levels (45). In humans, Wnt5a levels are higher in OSCC patients with smoking habits rather than in non-smoking patients, suggesting that nicotine is involved in the modulation of Wnt5a levels (45). Consistently, we observed transcriptional upregulation of Jun proto-oncogene, AP-1 transcription factor subunit (JUN) and nuclear factor of activated T cells (NFAT) forms, associated with PCP and Wnt/Ca2+ pathway respectively, in leukoplakia and early and late stage OSCC (Supplementary Figure S1). Indeed, JUN is a suitable prognostic biomarker of OSCC, especially in cases associated with HPV infection (46). Additionally, NFAT isoforms have been shown to promote oral carcinogenesis in hyperosmotic tumor conditions *in vivo* and *in vitro* (47, 48). Despite this may suggest a contribution of non-canonic Wnt pathways during oral carcinogenesis, more studies are required to validate that JUN and NFAT are activated in a Wnt-dependent manner in OSCC.

Interestingly, despite being described as activators of non-canonical Wnt pathways, Wnt7a and Wnt7b promote oral carcinogenesis in a canonical Wnt/β-catenin manner. For instance, overexpression of Wnt7a *in vitro* triggers nuclear translocation of β-catenin and enhanced migratory capacity of OSCC cells (49). Wnt7a expression also correlates with the degree of tumor differentiation in patients with OSCC (49). Consistently, a TCGA (The Cancer Genome Atlas) database analysis concluded that Wnt7a and Wnt7b levels correlate with the histological tumor differentiation of OSCC patients (50, 51). Wnt7b levels increase in dysplasia and OSCC in rats treated with 4NQO, leading to exacerbated proliferation and migration (50). Regulation of Wnt7b levels in OSCC are in part explained by downregulation of miRNAs miR329 and miR410 (52). Our transcriptomic analysis of the GEO database (GSE85195) shows that Wnt7a and Wnt7b mRNA levels are significantly increased in leukoplakia and early stage OSCC (Figure 1B). Wnt7b mRNA levels are also upregulated in late stage OSCC (Figure 1B). These results for Wnt5a and Wnt7b are consistent with a previously reported RNA-seq analysis (GSE70666) and validated by qRT-PCR in four OSCC patients (53). Collectively, this supports the information retrieved from the literature. Notwithstanding, there is a study that describes a non-canonical Wnt pathway activation by Wnt7a in OSCC carcinoma associated fibroblasts (CAFs), leading to increased cell migration of OSCC cells, which suggests a partial contribution of non-canonic Wnt pathway in oral carcinogenesis (54).

Wnt16 is a Wnt ligand that can activate both canonical and non-canonical Wnt pathways (55). Analysis of the GEO database (GSE85195) shows transcriptional upregulation of Wnt16 in leukoplakia and early and late stage OSCC (Figure 1B). To date, there is no evidence of the role of Wnt16 in oral carcinogenesis. However, it has been observed that Wnt16 KO mice display reduced serum 25(OH)D3 (calcidiol, precursor of the active form of Vitamin D, known as calcitriol) levels compared to WT littermates (56). Since patients with OPMD diagnosed with oral dysplasia display lower 1,25-(OH)2D3 (calcitriol), and 1,25-(OH)2D3 supplementation reduces β-catenin-dependent migration of dysplastic cells *in vitro* (57), future studies should evaluate whether Wnt16 is required for preventing the acquisition of malignant traits in Wnt/β-catenin. Another possibility is that Wnt16 is behaving as an inhibitor of excessive Wnt/β-catenin activation driven by other canonic Wnt ligands, as seen during dental pulp cell differentiation (58). Whether this happens in oral carcinogenesis is not known yet.

Activation of the Wnt/β-catenin pathway ultimately depends on stabilization and nuclear translocation of β-catenin. β-catenin is stabilized and drawn from the cell membrane in multiple well-differentiated OSCC cell lines (59). Similar results were obtained in a systematic review, where after meta-analysis of 41 studies, authors concluded that loss of β-catenin membrane localization is statistically associated with poorer overall survival of patients with OSCC (60). Advanced T and N status are also significantly associated with loss of β-catenin membrane localization (60). Histological grade (moderately/poorly-differentiated OSCC) is associated with aberrant upregulation of nuclear and cytoplasmic β-catenin (60). Consistently, moderately/poorly-differentiated OSCC displays reduced expression of membrane E-cadherin and increased expression of CCND1, a downstream target of transcriptionally active β-catenin (61). Interestingly, our GEO database (GSE85195) analysis concluded that E-cadherin is transcriptionally upregulated in early and late stage OSCC, suggesting that reduced expression of membrane E-cadherin in OSCC could be due to increased degradation or altered cellular localization, but not due to reduced synthesis (Figure 1C).

Dysregulation of components of the β-catenin destruction complex could also increase Wnt/β-catenin activity in cancer. The destruction complex is composed by the scaffold protein AXIN, which serves as a platform for the binding of other proteins, the integral protein APC that binds β-catenin, and two kinases: casein kinase 1 alpha 1 (CSNK1A1/CK1*α*) and glycogen synthase kinase 3 beta (GSK3B/GSK3β) (35). Once bound to APC, β-catenin experiences a sequential phosphorylation, first by CK1*α* at Ser-45 and then by GSK3B at Ser-33/Ser-37/Thr-41 (35). Phosphorylated β-catenin is targeted for proteasomal degradation (36).

Studies regarding transcriptional modulation of β-catenin destruction complex components are two decades old. Altered methylation of APC, GSK3B and AXIN is observed during oral dysplasia, before OSCC development (62, 63). After OSCC is established, the mRNA levels of APC severely drop with the differentiation status, suggesting a role of APC on controlling β-catenin levels in OSCC (62). Indeed, an aberrant nuclear distribution of APC protein is observed in OSCC, compared to normal and dysplastic oral tissues (62). Additionally, AXIN experiences truncation by inserting a thymine in codon 250, increasing the levels of β-catenin in *in vitro* and *ex vivo* models of OSCC (63). Mutation of AXIN also provokes the re-localization of β-catenin from the cell membrane towards the cytoplasm (63). However, conflicting results on mutations and transcriptional regulation of β-catenin destruction complex components have been reported (64).

By using the GEO database (GSE85195), we observed that APC mRNA levels are upregulated in leukoplakia and downregulated in late stage OSCC (Figure 1D). Additionally, transcriptional levels of GSK3B and AXIN are significantly higher than normal oral mucosa during leukoplakia, and early and late stage OSCC (Figures 1E,F). Similar results for AXIN transcriptional upregulation in OSCC have been reported elsewhere (65). Studies indicate that, compared to well and moderately differentiated OSCCs, poorly differentiated OSCCs display reduced protein levels of APC, AXIN and GSK3B, suggesting an increased β-catenin stabilization (41). During oral dysplasia, β-catenin is stabilized and translocated into the nucleus, while during OSCC β-catenin is drawn from the cell membrane (bound to E-cadherin) to the cytoplasm (66). It is feasible that contradictory results between the transcriptional expression from GEO database (GSE85195) and the reported protein levels of APC, GSK3B and AXIN, at least in leukoplakia and early stage OSCC, are due to the sequestration of these proteins in early endosomes, leading to stabilization of β-catenin and augmented TCF/LEF-dependent transcription (66).

All these findings highlights the contribution of the different Wnt signaling pathways during oral carcinogenesis, especially the Wnt/β-catenin pathway, for which the molecular mechanisms in OSCC are more understood.

## 6 The role of Wnt pathway in periodontitis and how this associate with OSCC

**Figure 2:**
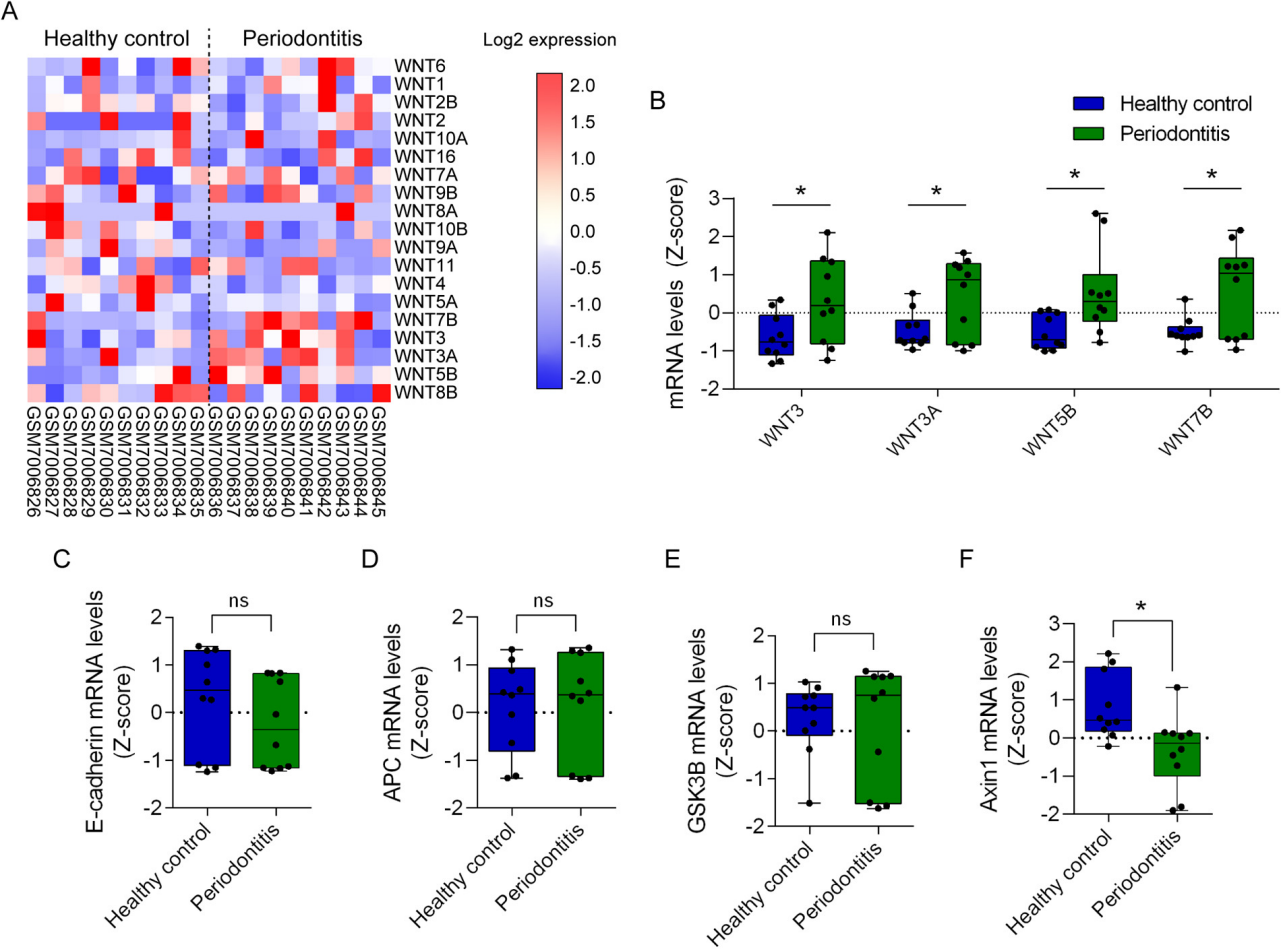
Transcriptomic analysis of Wnt ligands and components of β-catenin destruction complex in periodontitis. Transcriptomic datasets of 10 healthy controls and 10 periodontitis samples were retrieved from the GEO database GSE223924. Raw transcriptomic data from the database were processed and normalized to *Z*-scores to standardize the data and allow direct comparison of expression levels. **(A)** Transcriptional Wnt ligand expression was organized in heat maps according to their expression patterns. **(B)** *Z*-score of significantly upregulated Wnt ligands graphed and arranged according to the Ward hierarchical clustering. *T*-test was used for statistical analysis (*p < 0.05, vs. the *Z*-score of the corresponding healthy control). Additionally, expression data for critical components of the Wnt signaling pathway, including **(C)** E-cadherin (CDH1), **(D)** APC, **(E)** GSK3B and **(F)** Axin1, were graphed using GraphPad Prism. *T*-test was used for statistical analysis (*p < 0.05, vs. the *Z*-score of the corresponding healthy control).

To study the role of the Wnt pathway in periodontitis, we evaluated the transcriptomic expression of Wnt ligands and components of the β-catenin destruction complex in healthy and periodontitis patients (GEO database; GSE223924). Binary logarithm mRNA expression of Wnt ligands was graphically displayed using heat maps (Figure 2A). Transcriptomic *Z*-score analysis shows that among 19 Wnt ligands available, two canonical Wnt ligands (Wnt3 and Wnt3a) and two non-canonical Wnt ligands (Wnt5b and Wnt7b) are upregulated in periodontitis (Figure 2B). Of note, as previously indicated, despite Wnt7b is mainly associated with non-canonical Wnt activation, canonical Wnt/β-catenin pathway is activated by Wnt7b in OSCC (50).

Studies that have shown the relevance of Wnt ligands inducing the Wnt/β-catenin pathway during periodontitis progression, are mainly focused on Wnt3a. For instance, gingival tissues from patients with stage 3 periodontitis display higher Wnt3a, Wnt10b and nuclear β-catenin immunohistochemical staining, indicating the participation of the Wnt/β-catenin in periodontitis (7). Patients with advanced stages of periodontitis present elevated expression of Wnt5a as well (7). The experimental induction of periodontitis in rats, by drilling the first molar until the exposure of the pulp, is associated with higher Wnt3a and β-catenin (67). Expression of Wnt3a and β-catenin correlates with the volume of the periodontitis-induced lesion, suggesting that activation of the Wnt/β-catenin pathway associates with periodontitis progression (67). In a murine model of periodontitis, where a ligature is placed around the second molar, the increased nuclear β-catenin accumulation led to enhanced expression of TNF-α in macrophages (68). This indicates that during periodontitis a Wnt/β-catenin-dependent inflammation occurs. Consistently, clinical attachment level in patients with periodontitis is directly correlated with expression of β-catenin in the crevicular fluid (69). An *in vitro* study shows that human periodontal ligament fibroblasts treated with Wnt3a present reduced expression of alkaline phosphatase, a marker of early osteogenic differentiation (70). Since periodontitis is characterized by alveolar bone resorption, activation of the Wnt/β-catenin pathway may suppress the recovery of the alveolar bone during periodontitis. Finally, despite one study failed to observe changes in phosphorylated (inactive) β-catenin after stimulation with periodontal pathogen lipopolysaccharide (71), most studies highlight Wnt/β-catenin pathway as key for periodontitis progression.

Interestingly, the transcriptional upregulation of Wnt3, Wnt3a, Wnt5b and Wnt7b in periodontitis (GSE223924) was also observed in oral carcinogenesis (GSE85195) (Figures 1B, 2B). Among these four Wnt ligands, mRNA levels of Wnt3a, Wnt5b and Wnt7b are upregulated in leukoplakia and early stage OSCC, suggesting that if a causality is determined between periodontitis and OSCC, these ligands may participate in initiating malignant transformation of normal oral cells (Figures 1B, 2B). Accordingly, a single study has shown that *in vitro* infection of OSCC cell lines with a periodontal pathogen *Fusobacterium nucleatum* promotes the expression of Wnt5a and cisplatin chemoresistance (72). Despite no other links between upregulation of Wnt ligands during periodontitis and its role on oral carcinogenesis have been reported yet, other studies have inquired on whether β-catenin is activated in OSCC cells and tissues after periodontal pathogen challenge.

Oral carcinogenesis induced with 4NQO *in vivo* is significantly reduced if mice are germfree (73). Transcriptomic analysis revealed the enrichment of the Wnt/β-catenin pathway after 4NQO treatment (73). Despite authors do not report overrepresentation of periodontitis-related pathogens in 4NQO-induced microbiome, other studies have evaluated how these pathogens influence the activation of the Wnt pathways in periodontitis *in vitro*. In gingival epithelial keratinocytes, infection with *Porphyromonas gingivalis* results in proteolytic processing of E-cadherin, leading to nuclear translocation of β-catenin and increased the migratory capacity of the cells (74). Other periodontitis-related pathogen, such as *Fusobacterium nucleatum* induces proliferation of CAL27 cell lines, an *in vitro* OSCC model (75). Authors suggest that proliferation is due to the loss of E-cadherin at the membrane and stabilization of β-catenin (75). *Streptococcus gordonii*, on the other hand, precludes upregulation of zinc finger E-box binding homeobox 2 (ZEB2) in TIGK cells (Telomerase Immortalized Gingival Keratinocytes) (76). ZEB2, a protein that promotes epithelial mesenchymal transition (EMT), decreases the expression of E-cadherin (77). High levels of ZEB2 in OSCC are associated with increased metastatic potential and poorer survival of patients (78, 79). Importantly, given that no significant changes in E-cadherin mRNA levels were observed during periodontitis using the GEO database GSE223924, E-cadherin expression after periodontal pathogen exposure may not be explained by reduced transcription (Figure 2C). Nevertheless, it is feasible that ZEB2 interferes with the membrane localization of E-cadherin to activate β-catenin, as occurs in laryngeal squamous cell carcinoma (80). Whether, during oral carcinogenesis, periodontal pathogens can stabilize cytoplasmic and nuclear β-catenin in a ZEB2-dependent mechanism is not clear yet.

*In vitro* infection with *Porphyromonas gingivalis* also promotes a gingipain-dependent proteolytic processing of β-catenin and degradation of GSK3B (74, 81). Despite this cleaved form of β-catenin retains phosphorylation, suggesting that is transcriptionally active, further studies are required to postulate gingipain proteolysis as a non-canonical mechanism of β-catenin activation. Additionally, transcriptional expression analysis of components of the β-catenin destruction complex during periodontitis revealed no changes in APC and GSK3B expression (Figures 2D,E). A significant reduction of AXIN expression was also found (Figure 2F). Since the change in mRNA levels of APC, GSK3B and AXIN during periodontitis are not related to those observed during oral carcinogenesis (Figures 1C–F), it seems unlikely that transcriptional regulation of components of β-catenin destruction complex would be playing a role in periodontitis and oral carcinogenesis. However, whether altered localization of E-cadherin, APC, GSK3B and/or AXIN proteins occur during periodontitis to drive oral carcinogenesis remains unknown. This idea should be addressed in future studies. Finally, we did not find significant changes in transcriptional expression of JUN and NFAT isoforms, suggesting that non-canonic Wnt pathways do not participate in periodontitis (Supplementary Figure S2).

The results so far indicate that Periodontal pathogens may activate this pathway by increasing Wnt ligands, cleaving β-catenin via gingipains, or disrupting its interaction with E-cadherin at the cell membrane. Nevertheless, we cannot discard other mechanisms that might lead to Wnt/β-catenin pathway activation. Since all findings are circumstantial, more studies and systematic analysis are needed to properly understand the causal effects of this pathway in periodontitis and its possible relationship with OSCC.

## 7 Discussion

**Figure 3:**
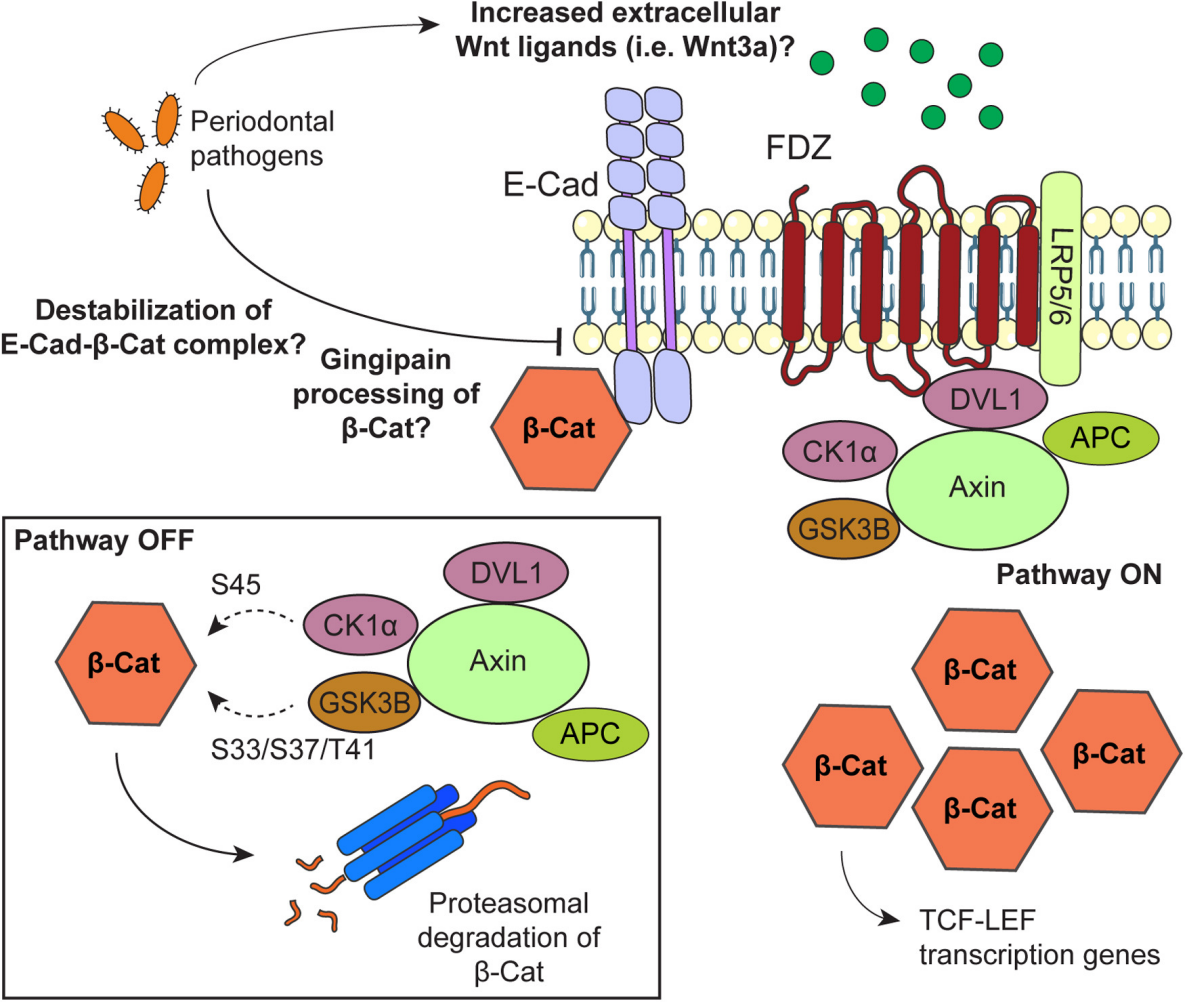
Possible mechanisms by which periodontal pathogens may promote activation of the Wnt/β-catenin pathway during oral carcinogenesis. When the Wnt/β-catenin is “off”, β-catenin is targeted for proteasomal degradation by a complex composed by dishevelled segment polarity protein 1 (DVL1), AXIN, APC regulator of WNT signaling pathway (APC), casein kinase 1 alpha 1 (CSNK1A1/CK1*α*) and glycogen synthase kinase 3 beta (GSK3B/GSK3β). To promote β-catenin degradation, CK1*α* and GSK3B phosphorylate β-catenin at S45 and S33/S37/T41, respectively. β-catenin is also found forming a complex with E-Cadherin at the cell membrane. To activate the Wnt/β-catenin signaling pathway, extracellular Wnt ligands bind to the Frizzled (FDZ) receptor and the LDL receptor related protein (LRP) 5/6 co-receptor. With this, the destruction complex is drawn from the cytoplasm and bound to the receptor at the cell membrane, leading to upregulation of β-catenin. Stable β-catenin translocates to the nucleus as a co-transcriptional factor of TCF/LEF genes. Possible mechanisms by which periodontal pathogens may activate the Wnt/β-catenin pathway include increasing the levels of Wnt ligands at the extracellular medium, a gingipain-dependent proteolytic processing of β-catenin, and disrupting the β-catenin-E-cadherin complex at the cell membrane.

In summary, studies suggest that the Wnt/β-catenin pathway participates in the progression of periodontitis. By analyzing the GEO databases GSE223924 and GSE85195 we found that the Wnt ligands Wnt3, Wnt3a, Wnt5b and Wnt7b are transcriptionally upregulated in periodontitis and OSCC, especially during early carcinogenic phases. Nevertheless, the information on how Wnt ligands could mediate oral carcinogenesis in periodontitis remains scarce. The link between periodontitis and OSCC by β-catenin activation is mainly derived from *in vitro* studies with periodontal pathogens. Thus, the modification of the oral microbiome seems promising to explain a possible Wnt-driven oral carcinogenesis by periodontitis. To this, activation of the Wnt/β-catenin may be associated with increased transcriptional expression of Wnt ligands, and/or altered localization or turnover of E-cadherin, APC, GSK3B and/or AXIN. Future studies using different *in vitro*, *in vivo* and *ex vivo* models are still required to validate these possibilities. These studies may also address whether changes in Wnt/β-catenin activation during different stages of periodontitis are linked to oral carcinogenesis. Current knowledge on how periodontitis may modulate the Wnt/β-catenin pathway is shown in Figure 3. During periodontitis, overexpression of extracellular Wnt ligands may activate Wnt pathway to promote oral carcinogenesis. Together, Figures 1, 2 and Supplementary Figures S1, S2, suggest that the Wnt/β-catenin pathway, but not the non-canonic Wnt pathways, is associated with periodontitis-induced OSCC. Our observations are supported by the studies retrieved from literature. Other possibilities, which also highlights the role of the Wnt/β-catenin pathway, include the destabilization of the interaction between E-cadherin and β-catenin by periodontal pathogens and a controversial way of β-catenin activation by a gingipain-dependent proteolytic processing. More studies should address whether these mechanisms could drive periodontitis-induced OSCC.

The molecular interplay between periodontitis and OSCC is clinically relevant. Without disregarding the multiple pathways that converge during oral carcinogenesis, a reductionist model explaining the relationship between some of the risk factors could be extremely useful for developing new pharmacological strategies. During the last decade several Wnt pathway interventions have been developed for treating different types of cancer, including breast, nasopharyngeal, and renal cell carcinoma, among others (82–85). From these interventions, the most promising for oral cancer treatment is the inhibition of Wnt ligand secretion (6, 66, 86). Clinical trials are now testing the effectiveness of O-acyltransferase Porcupine (PORCN) inhibitors, like WNT-C59 and LGK974 (patented by Novartis) for early OSCC (6, 86). Inhibition of Wnt ligand secretion is a bulk mechanism that may affect all Wnt pathways simultaneously, but evidence from *in vitro*, *in vivo* and *ex vivo* models of oral dysplasia and early OSCC stages shows that this intervention is mainly affecting Wnt/β-catenin pathway (6, 86, 87). Despite we found upregulation of JUN and NFAT isoforms (Supplementary Figure S1), more studies are needed to determine whether PORCN inhibitors preclude activation of non-canonical Wnt pathways to prevent oral carcinogenesis. Transcriptomic analysis during treatment with PORCN inhibitors may shed light on this (88). Limitations of this study are the lack of literature data regarding the follow up of patients with periodontitis and development of OSCC. Thus, with the information available it is possible to propose a correlation between periodontitis, OSCC and the Wnt/β-catenin pathway, but not a cause-effect link between them yet. Given that only *in vitro* studies have approached the latter, if this cause-effect link is proven to occur, more studies would be required to address this in a pathophysiological context.

Considering that Wnt pathway may link periodontitis and OSCC, novel Wnt-based pharmacological approaches for preventing oral carcinogenesis by targeting periodontitis could be considered in the future. However, based on Figure 2 and Supplementary Figure S2, if PORCN inhibitors prevent periodontitis, effects would be more likely attributed to Wnt/β-catenin pathway than non-canonic Wnt pathways. Collectively, this understanding opens a window to prevent oral carcinogenesis by treating the molecular changes driven by periodontitis.
