# Supplementary material for: Evaluating the link between periodontitis and oral squamous cell carcinoma through Wnt/β-catenin pathway: a critical review
Source: Front Oral Health. 2025 May 12;6:1575721. doi: 10.3389/froh.2025.1575721 (PMC12104182; doi:10.3389/froh.2025.1575721)
Supplement: Supplementary file 1 [file Datasheet1.docx]

**Supplementary Methods**

Transcriptomic analysis of GSE223924 and GSE85195 datasets

Briefly, GSE223924 dataset includes transcriptomic data of patient samples including 10 normal gingival tissue and 10 samples diagnosed with periodontitis. In contrast, GSE85195 dataset comprises gene expression profiling data from oral leukoplakia (OPL) and early-stage oral squamous cell carcinoma (OSCC) including RNA profiles from 15 OPL samples, 34 OSCC samples, and 1 independent control tissue sample from a healthy donor. Method description from published dataset reports a total RNA extraction, followed by RNA sequencing and gene expression analysis using high-throughput sequencing methods.

Raw transcriptomic data were downloaded from the GEO repository as normalized expression values. To ensure consistency across datasets and enable meaningful comparisons, all expression data were subjected to additional normalization using Z-score transformation. By applying this normalization strategy, we aimed to mitigate potential technical biases and emphasize biologically relevant expression patterns that may distinguish the experimental groups included in each dataset.

GSE85195

Bhosale PG, Cristea S, Ambatipudi S, Desai RS et al. Chromosomal Alterations and Gene Expression Changes Associated with the Progression of Leukoplakia to Advanced Gingivobuccal Cancer. *Transl Oncol* 2017 Jun;10(3):396-409. PMID: [28433800](https://www.ncbi.nlm.nih.gov/pubmed/28433800)

Inchanalkar M, Srivatsa S, Ambatipudi S, Bhosale PG et al. Genome-wide DNA methylation profiling of HPV-negative leukoplakia and gingivobuccal complex cancers. *Clin Epigenetics* 2023 May 27;15(1):93. PMID: [37245006](https://www.ncbi.nlm.nih.gov/pubmed/37245006)

GSE223924

Oh JM, Kim Y, Son H, Kim YH, Kim HJ. Comparative transcriptome analysis of periodontitis and peri-implantitis in human subjects. J Periodontol. 2024 Apr;95(4):337-349. doi: 10.1002/JPER.23-0289. Epub 2023 Oct 3. PMID: 37789641.
