# Supplementary material for: Evaluating the link between periodontitis and oral squamous cell carcinoma through Wnt/β-catenin pathway: a critical review
Source: Front Oral Health. 2025 May 12;6:1575721. doi: 10.3389/froh.2025.1575721 (PMC12104182; doi:10.3389/froh.2025.1575721)
Supplement: Supplementary file 2 [file Table1.docx]

**Table 1. Classification of Wnt ligands**

| **Canonic Wnt ligands** | **Non-canonic Wnt ligands** |
| --- | --- |
| Wnt1 | Wnt4 |
| Wnt2 | Wnt5a |
| Wnt2b | Wnt5b |
| Wnt3 | Wnt6 |
| Wnt3a | Wnt7a |
| Wnt8a | Wnt7b |
| Wnt8b | Wnt11 |
| Wnt10a |  |
| Wnt10b |  |
