# Supplementary figures and images for: Evaluating the link between periodontitis and oral squamous cell carcinoma through Wnt/β-catenin pathway: a critical review
Source: Front Oral Health. 2025 May 12;6:1575721. doi: 10.3389/froh.2025.1575721 (PMC12104182; doi:10.3389/froh.2025.1575721)

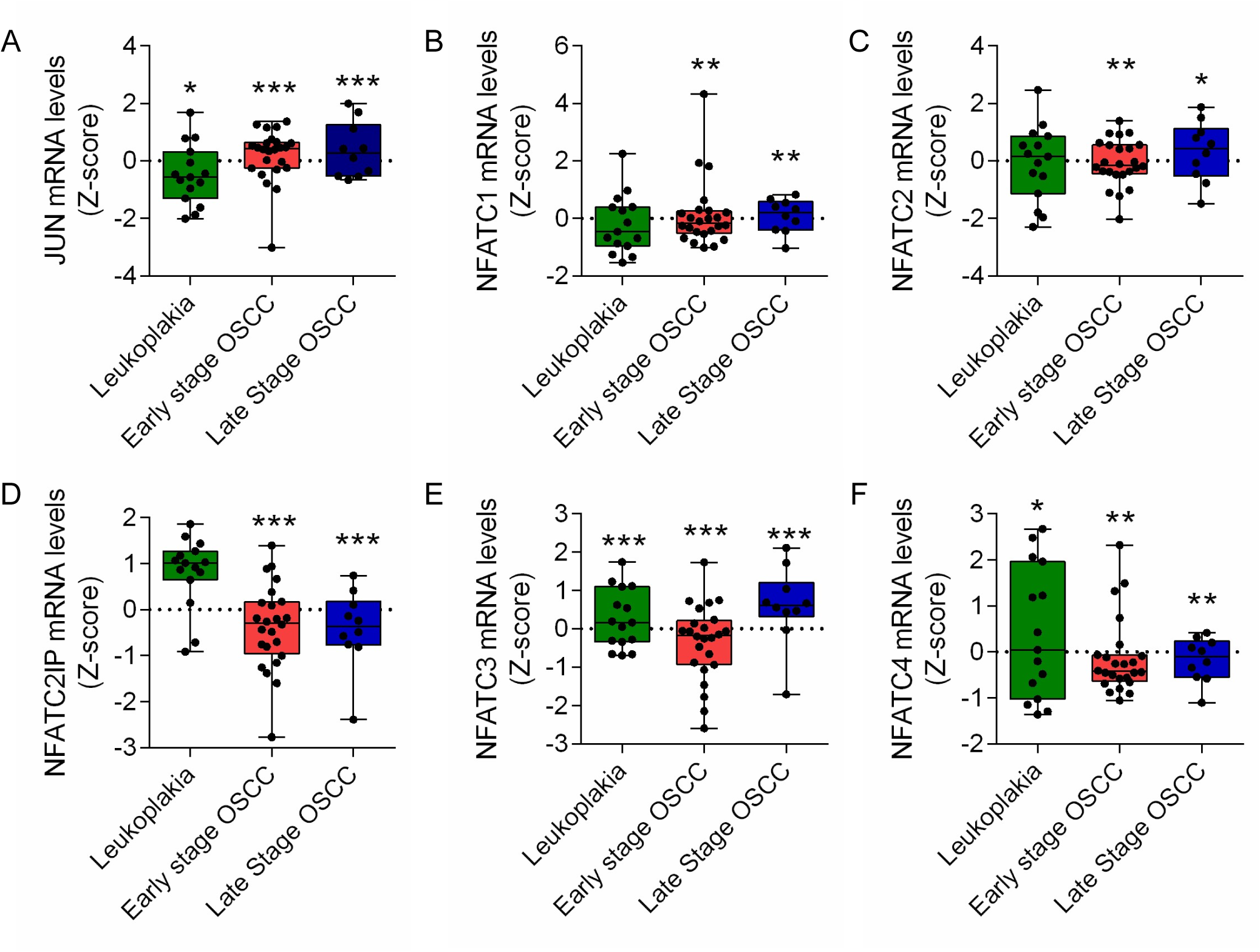

Supplement: Supplementary file 3 [file Image1.tif]

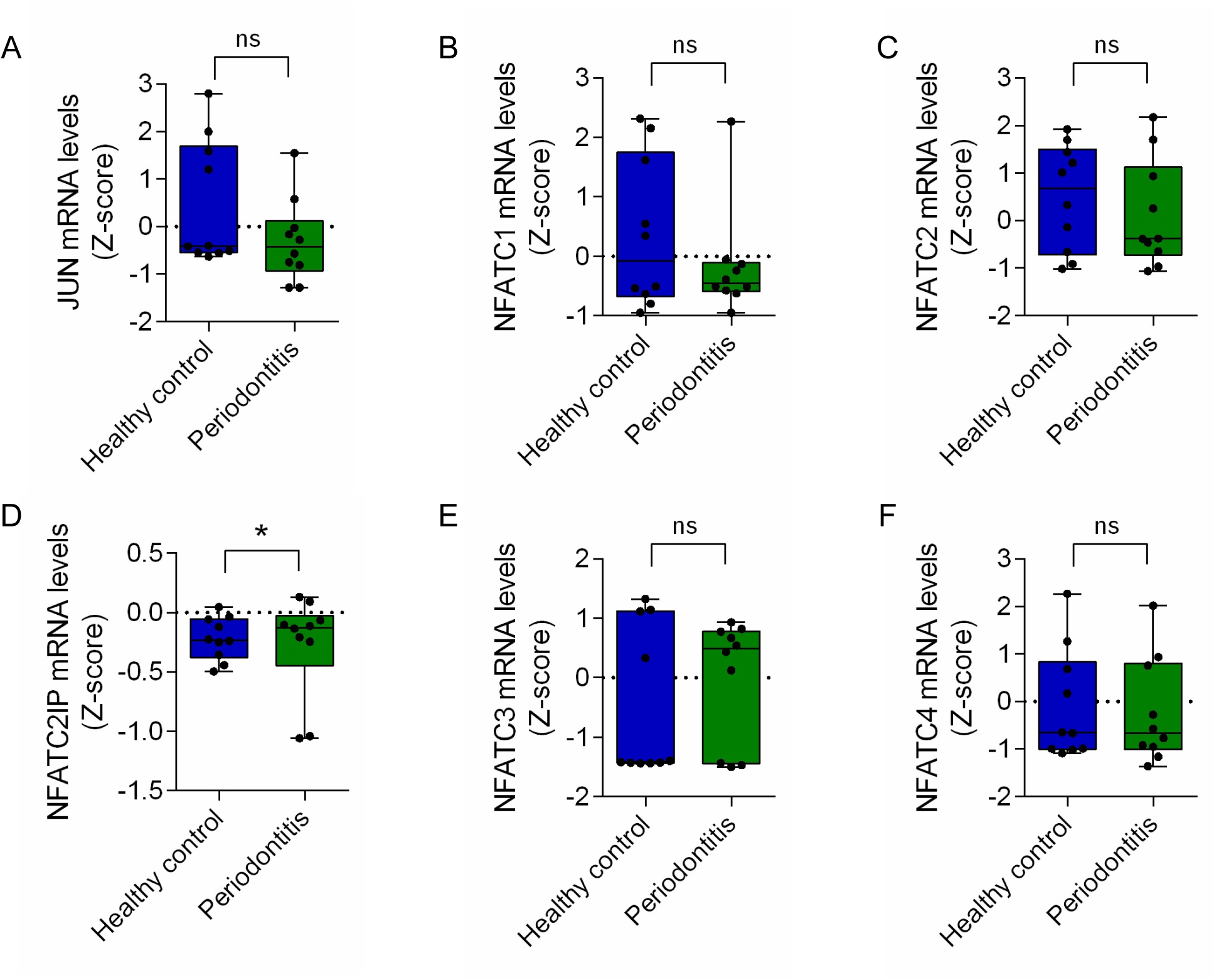

Supplement: Supplementary file 4 [file Image2.tif]
